# Microphysiological systems for human aging research

**DOI:** 10.1111/acel.14070

**Published:** 2024-01-05

**Authors:** Seungman Park, Thomas C. Laskow, Jingchun Chen, Prasun Guha, Buddhadeb Dawn, Deok‐Ho Kim

**Affiliations:** ^1^ Department of Mechanical Engineering University of Nevada, Las Vegas Las Vegas Nevada USA; ^2^ Department of Medicine Johns Hopkins University School of Medicine Baltimore Maryland USA; ^3^ Nevada Institute of Personalized Medicine University of Nevada, Las Vegas Las Vegas Nevada USA; ^4^ School of Life Sciences University of Nevada, Las Vegas Las Vegas Nevada USA; ^5^ Department of Internal Medicine, Kirk Kerkorian School of Medicine University of Nevada, Las Vegas Las Vegas Nevada USA; ^6^ Department of Biomedical Engineering Johns Hopkins University Baltimore Maryland USA; ^7^ Center for Microphysiological Systems Johns Hopkins University Baltimore Maryland USA

**Keywords:** age‐related changes, age‐related diseases, aging, aging phenotypes, microphysiological systems

## Abstract

Recent advances in microphysiological systems (MPS), also known as organs‐on‐a‐chip (OoC), enable the recapitulation of more complex organ and tissue functions on a smaller scale in vitro. MPS therefore provide the potential to better understand human diseases and physiology. To date, numerous MPS platforms have been developed for various tissues and organs, including the heart, liver, kidney, blood vessels, muscle, and adipose tissue. However, only a few studies have explored using MPS platforms to unravel the effects of aging on human physiology and the pathogenesis of age‐related diseases. Age is one of the risk factors for many diseases, and enormous interest has been devoted to aging research. As such, a human MPS aging model could provide a more predictive tool to understand the molecular and cellular mechanisms underlying human aging and age‐related diseases. These models can also be used to evaluate preclinical drugs for age‐related diseases and translate them into clinical settings. Here, we provide a review on the application of MPS in aging research. First, we offer an overview of the molecular, cellular, and physiological changes with age in several tissues or organs. Next, we discuss previous aging models and the current state of MPS for studying human aging and age‐related conditions. Lastly, we address the limitations of current MPS and present future directions on the potential of MPS platforms for human aging research.

## Graphical abstract

Age is one of the risk factors for many diseases, and enormous interest has been devoted to aging research. A human aging model using microphysiological systems (MPS) can provide a predictive tool to understand the molecular and cellular mechanisms underlying the human aging process and age‐related diseases. Here, we provide a review on the application of MPS in aging research.

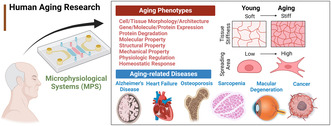

**Abbreviations**
2D: two‐dimensional
3D: three‐dimensional
Akt: Protein kinase B (also known as PKB)
AMP: Adenosine monophosphate
AMPK: AMP‐activated protein kinase
APAP: Acetaminophen
ATP: Adenosine triphosphate
BBB: Blood‐brain barrier
CK: Creatine kinase
CO2: Carbon dioxide
DNA: Deoxyribonucleic acid
EB: Evans blue
ECM: Extracellular matrix
FA: Focal adhesion
FDA: Food and drug administration
HepG2/C3A: Hepatocellular carcinoma
HGF: Hepatocyte growth factor
HGPS: Hutchinson‐Gilford progeria syndrome
hiPSC: Human induced pluripotent stem cells
hiPSC‐EC: Human induced pluripotent stem cell‐derived endothelial cell
hiPSC‐RPE: Human induced pluripotent stem cell‐derived retinal pigment epithelium
HSC: Hematopoietic stem cell
IL‐10: Interleukin‐10
iPSC: Induced pluripotent stem cells
MEMS: Micro‐electromechanical systems
MIMIC: Micromolding in microcapillaries
MPS: Microphysiological systems
MSC: Mesenchymal stem cell
mTOR: Mammalian target of rapamycin
NAD: Nicotinamide adenine dinucleotide
NaF: sodium fluorescein
ND: Neurodegenerative disease
NEJ: Neuroeffector junction
NF‐κB: Nuclear factor kappa B
NMJ: Neuromuscular junction
NSC: Neural stem cell
OcC: Organs‐on‐a‐chip
PDMS: Polydimethylsiloxane
PI3K: Phosphoinositide 3‐kinase
PR: Photoresist
Rho: Ras homolog
RhoA: Ras homolog family member A (RhoA)
RM: Replica molding
ROS: Reactive oxygen species
SAMIM: Solvent‐assisted micromolding
SASP: Senescence‐associated secretory phenotype
SIRT: Sirtuin
SMSC: Skeletal muscle stem cell
TAZ: Transcriptional coactivator with PDZ‐binding motif
TGF: Transforming growth factor
UV: Ultraviolet
Wnt: Wingless and int‐1
Wnt3a: Wnt family member 3a
YAP: Yes‐associated protein
μCP: Microcontact printing
μTM: Microtransfer molding

## 1 INTRODUCTION

Age is a major risk factor for numerous chronic conditions, including cancer, cardiovascular disease, neurodegenerative diseases (NDs), sarcopenia, osteoporosis, and kidney failure (Atella et al., 2019; Park, Jung, et al., 2020; Park & Kim, 2022; Sanchez et al., 2022). Damage to proteins and other biomolecules accumulates as people age, which results in biological deteriorations, such as genomic instability, mitochondrial dysfunction, or telomere attrition (López‐Otín et al., 2013, 2023) in cells, tissues, and organelles. A detailed understanding of specific aging processes and mechanisms is essential for developing therapeutic strategies for age‐related disorders.

**FIGURE 1:**
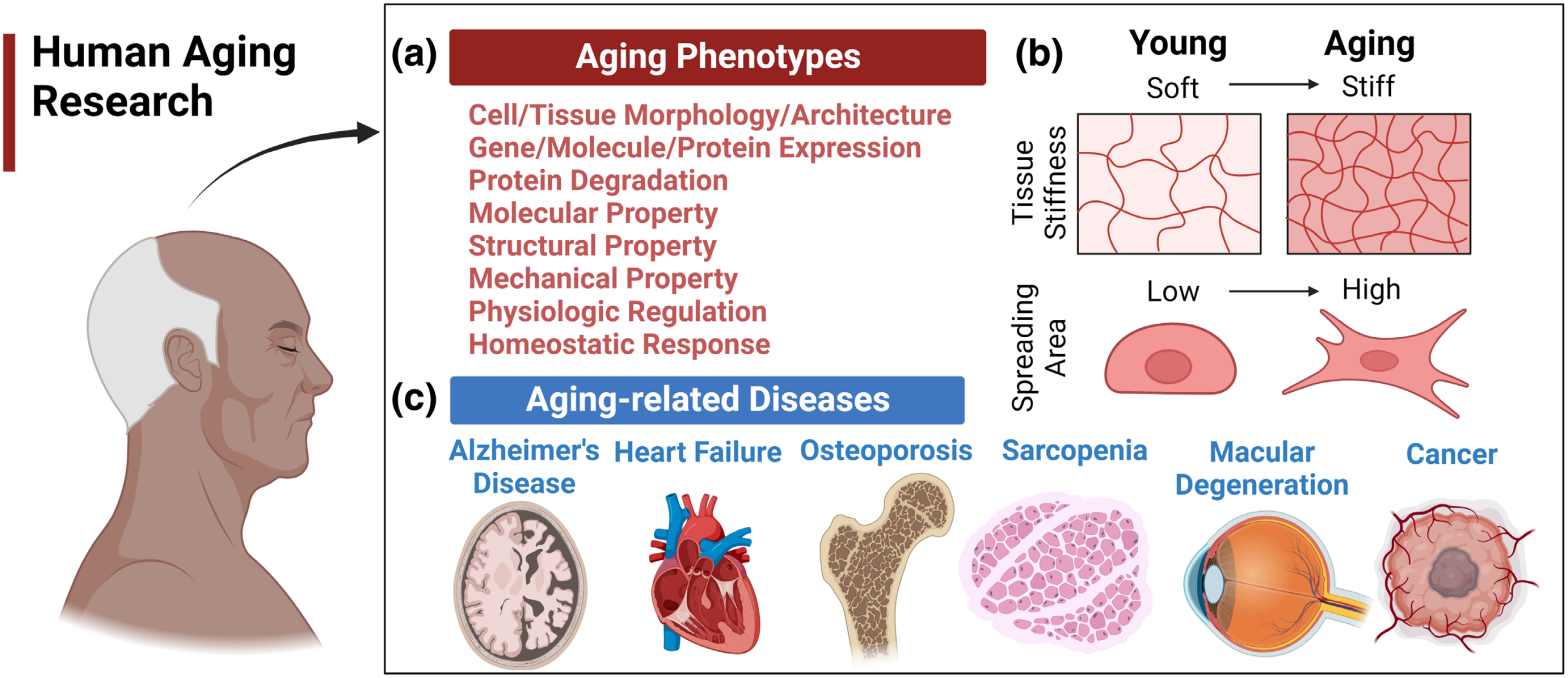
Types of aging studies. (a) Age‐related phenotypes and age‐related diseases. Representative phenotypes of normal aging include changes in cell/tissue architecture, abnormal RNA or protein expression, a decline in various functional properties, such as molecular, structural, or mechanical properties, altered physiologic rhythms, loss of complexity, and homeostenosis. (b) Examples of age‐related changes in cellular and tissue levels. At the cellular level, cell morphology is changed in the aging process with a higher spreading area. At the tissue level, the stiffness of skin tissue is known to increase significantly during aging. (c) Age‐related diseases at the organ levels. Examples of age‐related diseases are Alzheimer's disease, cardiovascular disease, cancer, osteoporosis, and so on.

Aging research has been focused on two major areas: age‐related phenotypes and age‐related diseases (Naylor et al., 2013; Figure 1). Age‐related phenotypes denote characteristics of “normal” aging, including natural changes in various properties, morphology, or architectures of biological systems (Figure 1a,b). Skin wrinkling, poor vision, and disrupted circadian rhythms, for example, are included in these common aging features. Age‐related diseases are health conditions that manifest with greater prevalence in individuals as they advance in age (Figure 1c). Diseases including Alzheimer's disease, heart failure, osteoporosis, and cancer are commonly considered to be aging‐related (Guo et al., 2022). The study of aging phenotypes provides useful information to evaluate human aging processes, optimally developing advanced techniques to slow biological aging. On the other hand, age‐related diseases represent diverse maladies derived to some degree from aging‐specific phenotypes. The goal of studying age‐related diseases is to understand the molecular and cellular mechanisms underlying the diseases, thereby finding the precise diagnostic and therapeutic targets for disease control. It is hypothesized that some age‐related phenotypes (particularly the age‐related biological changes termed “pillars” or “hallmarks” of aging) may contribute mechanistically to the development and progression of multiple age‐related diseases.

To date, various model systems have been employed in aging research, ranging from non‐mammalian model organisms, two‐dimensional (2D) cell cultures, and three‐dimensional (3D) organoid systems, to mammalian models, as delineated in Table 1. Nevertheless, these model systems come burdened with significant constraints. They struggle to faithfully replicate the intricate structures and physiological intricacies of tissues and organs, raise ethical concerns, entail exorbitant costs, among other limitations—for a more comprehensive exploration of these advantages and drawbacks, please refer to Section 3.1. As an alternative to these conventional models, the concept of microphysiological systems (MPS), also known as organ‐on‐a‐chip (OoC), has emerged as a promising frontier in human aging research (Kim et al., 2009; Wu et al., 2020). According to the U.S. Food and Drug Administration (FDA), MPS aim to “replicate the functional characteristics of a specific human or animal tissue or organ, by subjecting cells to a microenvironment that emulates the crucial physiological elements essential for their normal function or representation of a pathophysiological state (Anon, 2021).” When synergistically integrated with cutting‐edge techniques such as tissue engineering, bioprinting, soft lithography, microfluidics, and micro‐electromechanical systems (MEMS), recent strides in MPS technology have shown remarkable potential in recapitulating the complex physiology and pathology of various tissues and organs, achieving unprecedented levels of resolution.

**TABLE 1:** Advantages and disadvantages of model systems to study aging and age‐related diseases.

System	Advantages	Disadvantages	Examples of aging model type
Non‐mammalian model organisms	Rapid generation time Cost‐effective Fast identification of proteins and molecular pathways Low cost and easiness for maintaining	Differing life histories, physiology, and complexity relative to mammalian models Difficulty in translating findings into the context of human physiology	*Caenorhabditis elegans* *Saccharomyces cerevisiae* *Drosophila melanogaster* *Nothobranchius furzeri* *Danio rerio*
2D cell cultures	Simple and high throughput Easy manipulation High reproducibility Feasible modeling of cellular/mechanical communications	Lack of emulating complex tissue architectures and environment Limited vascularization Limited modeling for human physiology	Endothelial cells Keratinocytes Fibroblasts Macrophages Microglia cells
3D organoid systems	Superior to cell culture method in terms of mimicking some critical characteristics of native tissues Excellent for drug screening Allowing for genetic and pharmacological manipulation High‐throughput screening Modeling organogenesis and patient‐derived organoids Facilitated manipulation Feasible modeling for human physiology Feasible modeling of cellular/mechanical communications	Poor performance in providing oxygen/nutrients Low reproducibility Lack of immune‐related components High variability from one lab to another	Dermis organoid Cardiac organoid Gastrointestinal organoid Articular chondrocyte matrix
Mammalian model organisms	Excellent to study different physiological and pathological conditions Physiologically relevant vascularization with immune system activity Modeling for human physiology	Costly and time‐consuming Difficulty recapitulating human physiology due to the epigenomic and genomic differences Limited manipulation Low reproducibility High environmental and life‐course heterogeneity High genetic homogeneity Low‐throughput screen	Mouse Dog Cats Sheep Rabbit Pig Naked mole rat
MPS	Compatible with organoid or cell culture systems High throughput, scalable, simple, cost‐effective, and user‐friendly Excellent performance for perfusing oxygen, nutrients, and chemicals Vascularization Excellent mechanical and optical properties of Polydimethylsiloxane (PDMS)	Difference in physiology between in vitro MPS and in vivo human/animal models Lack of multi‐organ/tissue systems due to the difficulty in appropriate scaling of organs or development of a universal medium Difficulty in interpreting the real biological phenomena due to the non‐selective binding of proteins, molecules, or drugs to the PDMS surface	Microfluidic device Micropillar Boyden chamber PDMS channel Hydrogel Glasses

While MPS have been used for studies of many tissues and organs, only a few studies have been performed on both age‐related phenotypes in biological systems and age‐related diseases. In this review, we discuss prospects for aging research using MPS. First, we review how the molecular, cellular, and tissue properties or functions change during the aging process. Next, the status of MPS is addressed for aging and age‐related disease research, including previous model systems and studies. Finally, we discuss the current limitations of MPS in studying aging and their potential as a model for future aging research.

## 2 AGE‐RELATED CHANGES IN THE HUMAN BODY

For MPS to contribute fully as an experimental system in the study of aging, researchers must apply knowledge of cellular, tissue, organ, and physiological function gained from years of research in 2D cell culture, and model organism systems, as well as studies of aging humans. Aging drives changes in physiological function across diverse scales, including genetic/epigenetic modifications, alterations in signaling pathways, and biochemical and biophysical changes in organelles, cells, tissues, and organs (Phillip et al., 2015). In this section, representative age‐related biological phenotypes are discussed with connections drawn to the design and implementation of MPS focused on aging. Whether utilizing primary cell cultures from older adult tissues or induced pluripotent stem cell (iPSC)‐derived cells, researchers should strive to demonstrate similar age‐related molecular and physiological phenotypes in MPS intended for aging research. For a more extensive discussion of the molecular and physiological phenotypes of human aging, the reader is referred to several excellent recent reviews (Khan et al., 2017; López‐Otín et al., 2023).

### 2.1 Age‐related changes at the molecular level

#### 2.1.1 Changes in molecular functions and properties

Aging induces substantial alterations to many intracellular functions and properties at the molecular level, signaling pathways, epigenetic, and genetic expression. The genomic deoxyribonucleic acid (DNA) can be damaged during aging (Parrinello et al., 2005). Epigenetically, local hyper‐ and hypomethylation occur in aging cells (Jones et al., 2015). In addition, protein oxidation, glycation, and misfolding increase during the aging process. The combination of protein oxidation and misfolding can cause proteolysis‐resistant aggregates, as well as lipid‐protein aggregates associated with dysregulation and dysfunction of the central nervous system and the pathogenesis of neurodegenerative diseases (NDs) (Caughey & Lansbury, 2003; Crabb et al., 2002). Age‐associated protein aggregates arise concurrently with and may contribute to impairment in macroautophagy (Aman et al., 2021). In the context of MPS for aging research, experimental designs can benefit from a range of genetic, pharmacological, or other experimental interventions that have been described in 2D culture and animal model systems to induce or inhibit these age‐related molecular phenotypes (Aman et al., 2021; Thompson et al., 2010; Yousefzadeh, Henpita, et al., 2021).

In addition to the intracellular components, functions and density of extracellular molecular components, such as collagen, cross‐linker, fibronectin, and matrix metallopeptidase (MMP), are significantly affected by aging and age‐related immune cell changes (Sutherland et al., 2023). Collagen is the most abundant protein in the human body and constitutes approximately 90% of the extracellular matrix (ECM) and 30% of all proteins (Burgeson & Morris, 2021; Gilkes et al., 2014). The amount of collagen decreases with age, leading to a change in the mechanical strength of tissues and organs. Laminin, an ECM component in the basement membrane, also undergoes age‐associated changes. Studies have illustrated that elderly individuals have a thinner basement membrane, partly due to the decrease in protein biosynthesis (Frantz et al., 2010; Kwak, 2013). Fibronectin, involved in cell adhesion, migration, and the organization of the ECM, shows an increase in mRNA and, thus, an increase in its biosynthesis (Labat‐Robert, 2004). Aging drives remarkable alterations in the function and expression of many other molecules, including increased levels of activated MMPs, plasminogen activator inhibitors, and elastase activity, along with decreased levels of cross‐linking content, tissue inhibitors of metalloproteinases (TIMPs), and glycosaminoglycans (GAGs). Collectively, these changes influence the functional properties and integrity of ECM within tissues (Callaghan & Wilhelm, 2008; Robert, 1998). Two of the plausible reasons for increasing stiffness with age may be the age‐associated non‐enzymatic glycation of ECM proteins, including collagen, crystallin, and elastin, and altered ECM deposition, structure, and degree of cross‐linking (Henderson et al., 2020; Park, 2022; Phillip et al., 2015; Singh et al., 2001). Though a detailed discussion of these changes is beyond the scope of this review, it should be noted that age‐related changes to the ECM entail the study of fibrosis, a major contributor to age‐related chronic diseases and impaired wound healing.

The ECM, therefore, represents an important, if complex, aspect of aging tissues and organs, which researchers should consider in both the design and interpretation of MPS for aging research (Kutluk et al., 2023). Studies in organoid systems have previously utilized ECM substrates modified to recapitulate age‐related changes, specifically glycation, as well as using decellularized ECM from older humans or aged mammalian model organisms (Hu et al., 2018). The polydimethylsiloxane (PDMS) utilized in many MPS designs has multiple favorable properties (including tunable stiffness, optical transparency, and favorable cost). However, PDMS is currently limited by the surface hydrophobicity and associated non‐specific binding of small molecules and proteins, resulting in poor adhesion between PDMS and ECM. In addition, injecting high concentrations of ECM components, such as collagen, into microchannels is extremely challenging due to the high viscosity. These features can make the incorporation of certain ECM biophysical and biochemical properties more difficult. Methods to further integrate ECM and MPS systems are an active area of research (Kutluk et al., 2023).

#### 2.1.2 Changes in signaling pathways

During aging, signaling transduction and pathways are gradually and drastically altered, thereby affecting the cytoskeleton, organelles, and cell dynamics (Pelissier et al., 2014; Wu et al., 2011). MPS have favorable characteristics for studying pathways that are altered by 2D culture conditions or are challenging to measure in situ in animal models. One example of this is the capacity of MPS to simulate mechanobiological cues that can allow in vitro cells to more closely recapitulate the behaviors and signal responses that arise in vivo (Thompson et al., 2020). In aged cells, sensing and transducing of biomechanical and biochemical signals are impaired (Bajpai et al., 2021). Since cellular mechanotransduction is highly dependent on the state and dynamics of the cytoskeleton (e.g., actin filament), age‐related changes in the function and density of the cytoskeleton can cause the dysregulation of mechanosignaling pathways, thus leading to abnormal cellular force generation, functions, and related biological processes (Bajpai et al., 2021). Cells sense external signals and convert them into biochemical signals through structural molecules, such as integrin and focal adhesions (FAs) (Hoffman et al., 2011). During aging, the distribution and activation of FAs are notably changed. FAs are accumulated in the perinuclear region in aged cells (Arnesen & Lawson, 2006; Rice et al., 2007). Some FA‐related proteins and signals also appear to increase with age (Rice et al., 2007). Aspects of mechanotransduction correlate directly to other age‐related phenotypes such as the maintenance of a senescent cell phenotype. In a 2D cell culture model of aging that utilized human fibroblasts, high cell passage number activated mechanosensitive signaling pathways related to Ras homolog (Rho) GTP CDC 42, Rac1, and caveolin‐1. Caveolin‐1 knockout could convert these senescent cells toward a spindle‐shaped morphology more typical of young cells. This reversion to a younger phenotype was associated with reduced FAs and actin stress fiber formation (Cho et al., 2004).

Also, yes associated protein (YAP) and transcriptional coactivator with PDZ‐binding motif (TAZ) have been identified as master regulators of mechanical signaling between cells and their surrounding ECM using mechanotransduction. A recent study in mice demonstrated an age‐associated decrease in YAP/TAZ activity in stromal cells and downstream activation of cGAS‐STING, promoting senescent cell characteristics including senescence‐associated secretory phenotype (SASP) gene expression (Sladitschek‐Martens et al., 2022). This decreased YAP/TAZ activity appeared to be independent of the canonical upstream Hippo signaling pathway and adds to prior reports of age‐associated changes in YAP/TAZ activity regulated by the Hippo pathway (Chen et al., 2018; Nardone et al., 2017; Sun et al., 2014). In aging/aged cells, abnormal signal transduction leads to impaired mechanical force and properties (Hoffman et al., 2011). Specifically, overactivated Ras homolog family member A (RhoA) signaling in aged cells can lead to abnormal stiffness and tension (Sakuma et al., 2008). Moreover, YAP/TAZ can move from the nuclear to the cytoplasm of aging cells, including human mammary epithelial cells, dermal fibroblasts, and adipose endothelial cells. In this way, target genes are downregulated (Mammoto et al., 2019; Tsikitis et al., 2018). Other studies have exhibited contradictory results. Stiffness of tissues such as skin or liver tends to increase with age, and the increased stiffness promotes nuclear localization of YAP/TAZ of aged mouse muscle fibroblasts (Stearns‐Reider et al., 2017). The difference in terms of nuclear localization of YAP/TAZ during aging may depend on the specific tissue/organ context or be due to increasing heterogeneity in aging cells and ECM. Further studies are needed to elucidate the discrepancy (Sliogeryte & Gavara, 2019). MPS might contribute to this and other aspects of aging mechanobiology given features such as the ability to tune substrate stiffness as well as modulate other relevant biomechanical cues.

It is not surprising, given the entwined nature of proposed aging biological signaling pathways, that integrins also signal via the phosphoinositide 3‐kinase (PI3K)/protein kinase B (Akt)/mammalian target of rapamycin (mTOR) signaling pathway, which regulates cell survival, growth, proliferation, cell migration, and angiogenesis, among other functions. Its activation can be mediated by mechanical loading. Downstream of Akt, mTOR is a serine/threonine protein kinase capable of forming either of two distinct complexes, mTORC1 or mTORC2 (Miyazaki et al., 2008; Papadopoli et al., 2019). mTORC1 activates downstream pathways such as nuclear factor kappa B (NF‐κB), involved in inflammation, as well as other pathways related to metabolism, lipogenesis, autophagy inhibition, and apoptosis (Pan & Finkel, 2017).

In addition to the role of Akt/mTOR in mechanosensing, mTOR is also a core protein for nutrient sensing. It is activated in response to growth factors and abundant availability of amino acids. In this context, mTOR, which promotes some pathways associated with adverse age‐related physiology and disease, is counterposed to AMP‐activated protein kinase (AMPK) an energy sensor of intracellular adenosine triphosphate (ATP) levels that plays an essential role in adjusting metabolic energy (Yu et al., 2021). AMPK is activated in response to nutrient scarcity and promotes autophagy both by activating kinase 1/2 (ULK1/2) and inhibiting mTOR, among other downstream effects, whose net result is increased nutrient availability, including an increase in nicotinamide adenine dinucleotide + (NAD+) (Li & Chen, 2019). NAD+ in turn promotes sirtuin (SIRT) pathway signaling, another key regulation pathway associated with longevity and health span. Studies have suggested a protective effect of sirtuins against a range of age‐related diseases such as cancer, diabetes, and NDs. SIRT1, especially, is implicated in processes including DNA repair, inhibition of inflammation, regulation of mitochondrial biogenesis, and stress resistance to ROS (Bernier et al., 2011; Bhatt & Tiwari, 2023; Brunet et al., 2004; Cohen et al., 2004; Liu et al., 2019). These age‐associated metabolic signaling pathways regulate not only nutrient sensing and response but also core aspects of stress response, cell survival, and inflammatory response. MPS may provide opportunities to clarify how perturbations to these age‐associated metabolic pathways affect physiologic function, as well as how MPS designed to mimic age‐associated physiology may feedback to affect these intracellular signaling pathways. In the context of organoid tissue culture, researchers previously fabricated iPSC‐derived liver tissue with knockdown of SIRT1 and demonstrated that SIRT1 deficiency induced fatty liver disease—the authors noted that this model, though partially successful, lacked important features of non‐alcoholic steatohepatitis in humans such as (a) increased collagen deposition and (b) changes to metabolic zonation. To better approximate human in vivo pathophysiology, the team proposed both the incorporation of additional cell subtypes (stellate cells) and the deployment of MPS to more finely control tissue oxygenation. This example underscores the potential for aging researchers to simultaneously refine the understanding of age‐related physiology and pathophysiology, while also refining the experimental systems used to study those processes (Collin de l'Hortet et al., 2019).

### 2.2 Age‐related changes at the levels of organelles, cells, and beyond

Mitochondria are membrane‐bound cell organelles that are highly complex and dynamic. Aging can alter various functions, organization, morphology, and bioenergetics of mitochondria (Seo et al., 2010). Studies have shown that elevated electron leakage, oxidative stress, and ROS occur with chronological age, leading to impaired efficacy of the electron transport chain (Massudi et al., 2012). Moreover, as an organism ages, ATP production, mitochondrial functions, and turnover (i.e., the balance between mitochondrial biogenesis and mitophagy) are reduced or diminished, mainly due to impaired mitochondrial degradation, biogenesis, or mitophagy (López‐Otín et al., 2013; Seo et al., 2010; Short et al., 2005; Terman et al., 2010). Other studies showed contradictory results that a decline in mitochondrial function or elevation of antioxidant defense is not associated with changes in life span or aging phenotype (Pérez et al., 2009; Trifunovic et al., 2004; Trifunovic & Larsson, 2008). It is known that mitochondria can adapt their functions and morphology in response to biomechanical and biochemical stimuli. However, these adaptations are diminished with age. For example, morphological plasticity and biogenetic capacity are known to decrease as a result of aging (Seo et al., 2010). When designing and interpreting results of MPS intended to study age‐associated mitochondrial changes, it will be important to consider the effects of cell culture and other perturbations, such as induced pluripotency, that may affect the fidelity of aging mitochondrial phenotypes (de Kok et al., 2021; Suhr et al., 2010).

Nuclear morphology and mechanics are influenced by aging. The nuclear shape of cells from aging tissues was found to be abnormal due to the loss of laminar integrity (Brandt et al., 2008; Scaffidi & Misteli, 2006). The nuclear volume in senescent cells tends to be larger compared to cycling cells (Kuilman et al., 2010; Swanson et al., 2013). Nuclear blebs, which form in areas where lamin fibers and lamina meshwork are not dense, are one of the hallmarks of senescence cells. The blebs can alter the 3D shape of the nuclei to a lobulated structure (Funkhouser et al., 2013; Goldman et al., 2004). Nuclear mechanics is tightly linked to cytoskeletal mechanics via lamin A/C (Isermann & Lammerding, 2013). Studies have demonstrated that cytoskeletal stiffness and RhoA activation are increased in aging cells, leading to increased nuclear stiffness and defective nuclear architecture (Mu et al., 2020).

Cell behaviors and functions change with age. Previous studies exhibited that aging decreases mobility and impairs the function of cells, thereby reducing the wound healing rate of diverse tissues such as skin or bone tissues (Ahmed et al., 2017; Bajpai et al., 2021; Ho et al., 2005). Aging has also been known to have a detrimental impact on diverse stem cells. For example, aging brings about deterioration of neural stem cell (NSC) proliferation and increased NSC senescence, which is associated with NDs, olfactory dysfunction, and spatial memory deficits (Enwere et al., 2004; Ming & Song, 2011). The capacity of mesenchymal stem cells (MSCs) from bone marrow and adipose tissue to control oxidative stress was substantially reduced with increasing age, which ultimately led to cell apoptosis, necrosis, and autophagy (de Barros et al., 2013; Haines et al., 2013; Stolzing et al., 2008). During aging, adult skeletal muscle stem cells (SMSCs) displayed a remarkable reduction in their regenerative capacity because of the buildup of the altered progeny (Brack et al., 2007; Brack & Muñoz‐Cánoves, 2016; García‐Prat et al., 2013). Aging influences the function and homeostasis of hematopoietic stem cells (HSCs), triggering cell cycle dysregulation and hematological malignancy (Pietras et al., 2011). These changes to cell behavior as well as many others offer contexts in which MPS may help researchers to situate cellular dysfunction within the wider context of tissue and organ function. Systems that integrate multiple cell types, tissues, and organ systems may be particularly well‐suited to improving our mechanistic understanding of the so‐called integrative hallmarks of aging, including chronic inflammation, stem cell exhaustion, and altered intercellular communication (López‐Otín et al., 2023).

Significant advancements have been achieved in the identification of individual genes, pathways, molecules, organelles, cells, and their interactions within the mechanisms that influence aging; however, there remains an outstanding challenge to integrate these mechanisms into a whole organism for understanding of aging from a complex systems perspective (Cohen et al., 2022). The complexity of whole‐organism biology may be particularly important for understanding multisystem aging phenotypes such as physical frailty and resilience (Fried et al., 2021; Scheffer et al., 2018). Until recently, dominant approaches to understanding the biology of aging were characterized by either bottom‐up or top‐down thinking. As an example of bottom‐up thinking, it was widely accepted that aging in organisms was primarily attributed to the cumulative damage from free radicals over time, as proposed by Harman's free radical theory of aging (Barja, 2002; Hekimi et al., 2011; Magalhaes & Church, 2006; Van Remmen et al., 2003). Conversely, from a top‐down perspective, aging itself can induce substantial alterations in ECM fiber orientation, density, cytoskeletal arrangement, and organelle composition, consequently leading to modifications in cellular signaling pathways and related gene expression (Bajpai et al., 2021). However, a wealth of studies has now highlighted that aging in organisms is the result of intricate networks and interactions among a multitude of organelles, molecules, and genes. This suggests that the entities we examine, such as molecules, organelles, and cells, are integral components of a larger interconnected system, where their behavior and functions cannot be fully comprehended without a thorough understanding of their interactions with other components at the systemic or whole‐organism level.

To address this requirement, the “complex systems” approach has been adopted, encompassing multiple hierarchical scales to attain a dynamic comprehension of the organism. Lipsitz (1992, 2002) demonstrated that the aging process is linked to a reduction in both functional and structural complexity within physiological processes. Their findings suggest that this diminishing complexity can impair an individual's capacity to withstand the stresses of daily life, consequently increasing the risk of disease and disability. As an example, the aging process entails a reduction in complexity within anatomical structures, such as the degeneration of intricate fractal‐like trabecular networks in bone, resulting in fractures or impairing the branching architecture of Purkinje fibers which are crucial for electrical conduction and the propagation of impulses within the heart's ventricular muscle. Consequently, this can contribute to the development of conduction diseases within the heart. In addition to the example mentioned above, the reduction in complexity within physiological systems due to aging is strongly linked to a wide range of diseases, including arrhythmias, cardiovascular conditions, and dementia (Lipsitz, 2002; Ma et al., 2021). In conclusion, to comprehend the aging process and its impact on physiology, it is imperative to integrate diverse mechanisms across multiple hierarchical scales, adopting a complex systems perspective. MPS are capable of modeling physiological perturbations, as well as interactions between multiple cell‐ and tissue‐types in a relatively high‐throughput and cost‐effective manner. This makes MPS a promising experimental platform both to study aging from a complex systems perspective, and to test interventions that target aging biology in the context of these complex interactions.

## 3 MICROPHYSIOLOGICAL SYSTEMS FOR STUDYING AGING PHENOTYPES AND AGE‐RELATED DISEASES

MPS are a cutting‐edge technology that utilizes microchips or microfluidic devices to simulate the function and pathologies of cells, tissues, or organs in the human body. These models can be leveraged to gain a better understanding of complex biological systems and disease mechanisms, as well as to test drug efficacy and toxicity.

As mentioned above, aging research can be categorized into two main areas: age‐related phenotypes and age‐related diseases. Age‐related phenotypes refer to the characteristics associated with normal aging, encompassing natural changes in various biological systems such as properties, morphology, or architectures. Age‐related diseases, on the other hand, are health conditions that become more prevalent as individuals grow older. In terms of mimicking complexity, the use of MPS is a promising method for conducting aging research, especially on recapitulating human tissues and organs. MPS provide a reproducible and high‐throughput system for modeling aging and human diseases and has the potential to simulate human tissues with fidelity, thus allowing us to study the effects of aging on individual parts of the human body. Currently, only a few studies have used MPS to recapitulate the complex aging environment for studying aging phenotypes and their related mechanisms rather than age‐related diseases. These studies have introduced aspects of biological aging at different scales—from cellular, tissue, and organ levels. In Section 3.1, we present illustrative examples of non‐MPS models and elaborate on the significance of MPS. In Sections 3.2 and 3.3, the design and fabrication methods commonly used for MPS are presented. In Section 3.4, we provide several examples demonstrating the application of MPS in the study of age‐related phenotypes, while Section 3.5 exhibits various examples of MPS usage in the study of age‐related diseases. Finally, in Section 3.6, we provide an extensive discussion of drugs utilized in MPS and potential drug candidates for aging research.

### 3.1 Previous model systems and the necessity of MPS

To study aging physiology/pathology and age‐related diseases, researchers have developed different model systems, including non‐mammalian model organisms, cell cultures, spheroids, and animal models (Table 1). The non‐mammalian model organisms include *Caenorhabditis elegans*, *Saccharomyces cerevisiae*, *Drosophila melanogaster*, and *Danio rerio*. For instance, the *C. elegans* model was adopted to study age‐dependent changes in the nucleus, including nuclear structure and mechanics (Haithcock et al., 2005). Results suggested that age‐related mechanical changes, such as softening of the nucleus, may trigger abnormal chromatin organization, genomic instability, and epigenetic defects (López‐Otín et al., 2013). Traditional 2D cell culture methods have also been widely utilized in modeling the aging environment. 2D cell culture methods are relatively easy to handle with relatively high consistency, reproducibility, and throughput. In vitro cell culture models have been used to study the fundamental physiology and pathology of NDs (Choi et al., 2016; Young & Goldstein, 2012) and the electrophysiology of human cortical neurons (Chun et al., 2021). However, there is a significant gap in the physiology between non‐mammalian model organisms or cells and complex in vivo human tissues and organs, with respect to architecture and function. The pitfalls of translating between non‐mammalian and mammalian aging models are underscored by a recent review demonstrating that the life‐extending effects of compounds in *C. elegans* and *D. melanogaster* are inconsistently conserved in mouse models (Bene & Salmon, 2023).

To address the limitations of the above two model systems, mammalian models, including non‐human primates, dogs, cats, sheep, rabbits, mini‐pigs, guinea pigs, and other small rodents, have been suggested for studying aging biology due to their similarity to humans in terms of anatomy, genetics, and physiology/pathology (Santulli et al., 2015). Many age‐related phenotypes have been analyzed using mouse models, including bladder function, behavior and grip, kidney function, hematology, immune function, glucose, insulin‐like growth factor (IGF‐1), body composition, cataracts, and lifespan (Ackert‐Bicknell et al., 2015; Heinze‐Milne et al., 2019; Levey et al., 2011; Newman et al., 2005; Wu et al., 2021). In addition, small rodents and swine models have been established for studying diverse aging‐associated diseases, such as Alzheimer's disease (Aguzzi & O'Connor, 2010; Oakley et al., 2006), progeria syndrome (Harkema et al., 2016), myocardial infarction (Coronel et al., 2007), and osteoporosis (Matsushita et al., 1986). Despite the advantages of animal models, some age‐related diseases, such as cardiovascular disease, Alzheimer's disease, and osteoporosis (Rangarajan & Weinberg, 2003; Vanhooren & Libert, 2013), which often occur in humans, may not occur in other animals, for example, mice. Moreover, key mediators, such as DNA repair, telomere length, and telomerase activity affecting aging, were found to be significantly different in their function between humans and mice.

Advanced tissue culture systems provide opportunities to address the limitations of 2D cell culture and animal models. Two important types of advanced tissue culture systems are MPS (the focus of the current review) and 3D organoids. Recently, evidence has demonstrated that organoid systems are a promising technology for modeling the fundamental aging process and for developing effective ways of treating age‐related diseases. Studies have found that organoid culture systems can recapitulate complex behaviors of cells and tissues as well as aging‐associated changes in functions. Some studies have showed that organoids have greater genomic stability and reproducibility than other model systems (Behjati et al., 2014; Blokzijl et al., 2016). For example, 3D organoids yielded enhanced gene expression and cellular functions, similar to physiological conditions from hepatocytes, chondrocytes, and mammary epithelial cells (Astashkina et al., 2012; Ben‐Ze'ev et al., 1988; Simian & Bissell, 2017). Moreover, scaffold‐free 3D neural organoids can promote the accumulation of amyloid aggregates and tauopathy, a phenomenon never seen in 2D cell cultures (Raja et al., 2016).

With the remarkable advance in organoid techniques, many different types of organs and tissues have been developed. Dos Santos et al. generated a human dermis organoid that could last for 120 days. With this organoid, they observed morphological changes with increased cellular senescence over time, which is consistent with those of chronological aging dermis tissue in vivo (Dos Santos et al., 2015). Lee et al. (2008) created a cardiac organoid chamber to measure pressure and ejection fraction. Gastrointestinal organoids were fabricated by several groups to study the aging microbiome and aging‐induced vulnerability to disease (Bartfeld et al., 2015; Heintz & Mair, 2014). Another organoid assay revealed that cell proliferation and stem cell function on the ECM from older individuals decline faster than those from young individuals (Gullapalli et al., 2005; Williams et al., 2014). Barbero et al. (2004) developed an aging articular chondrocyte matrix system, where they found age‐related function decline in tissue, such as matrix deposition. 3D organoid models, engineered by Lozito et al. (2013), were shown to be well fit for studying osteoarthritis.

Currently, pluripotent stem cells are frequently utilized as a source for organoid systems. Choi et al. developed a 3D human stem cell‐derived organoid model to mimic Alzheimer's disease. They discovered that neuronal maturation, as well as tauopathy, are enhanced in this model system, which was never observed in the animal model and 2D culture (Choi et al., 2014). Stem cell‐derived organoids also allowed researchers to study the age‐related changes in circadian rhythm (Moore et al., 2014). In addition, organoids are widely used to study the relationship between aging and stem cell research. For example, intestinal organoid research demonstrated that intestinal stem cell (ISC) function declines with age, where Wnt (wingless and int‐1) signaling is affected due to a deficiency of ISC niche from old mice (Nalapareddy et al., 2017). The addition of the Wnt3a (Wnt family member 3a) supplement restored ISC organoid formation and aged ISC growth. Organoids have also been adopted to study the relationship between caloric restriction and anti‐aging effects (Mattison et al., 2017; Yilmaz et al., 2012). Additionally, organoids have been proven to be excellent models for studying the relationship between aging and various age‐related diseases, including cancer (Kim et al., 2020), retinal disease (Fligor et al., 2018), cardiovascular disease (Cashman et al., 2016; Wang et al., 2014), brain disease (Lancaster et al., 2013; Mariani et al., 2015), or bone‐related diseases (Nguyen‐Ngoc et al., 2014). In an instance of cancer research, senescent fibroblasts in non‐malignant mammary organoids were shown to promote the invasion and proliferation of cancer cells with a decrease in epithelial differentiation (Parrinello et al., 2005). Despite a wealth of strengths, organoid systems have displayed some limitations, including high variation from lab to lab, low reproducibility, lack of immune‐related components, and poor performance in providing oxygen and nutrients.

As an alternative to these previous systems, the MPS have emerged as a promising technology for human aging studies (Kim et al., 2009; Wu et al., 2020). Such studies have been done in *diverse* ex vivo tissues and organs, including the heart (Aung et al., 2016; Criscione et al., 2023; Huebsch et al., 2016), kidney (Jang et al., 2017; Sciancalepore et al., 2014; Wilmer et al., 2016), lung (Bovard et al., 2018; Lagowala et al., 2021; Yang et al., 2018), liver (Beckwitt et al., 2018; Materne et al., 2013), brain (Brown et al., 2015; Deosarkar et al., 2015; Herland et al., 2016; Maoz et al., 2018), eye (Achberger et al., 2019; Bennet et al., 2018; Dodson et al., 2015), intestine (Kim et al., 2012; Miller et al., 2020; Steinway et al., 2020), neurovascular unit (Lyu et al., 2021), blood vessel (Park et al., 2010), and skin (Mohammadi et al., 2016; Ramadan & Ting, 2016; Wufuer et al., 2016). One of the most significant and pioneering studies which ignited the rapid development of MPS is the novel “lung‐on‐a‐chip,” invented by Huh et al. (2010), which mimics the complex alveolar‐capillary interface of the human lung for a nanotoxicology study. Using this microsystem, they examined the effects of mechanical strain on inflammatory responses to nanoparticles as well as the cellular uptake of nanoparticles. This study became the cornerstone for subsequent OoC studies.

### 3.2 Design of MPS platform

It is essential to consider several crucial parameters, such as geometry, flow conditions, and the transport of molecules or drugs when designing MPS (Tajeddin & Mustafaoglu, 2021). To date, various MPS designs, each with distinct geometrical features, have been developed to mimic different tissues or organs. Consequently, there is no universally standardized single design applicable to all MPS models. MPS in use typically exhibit sizes ranging from millimeters to submicrometers, with common shapes including circular and rectangular configurations (Damiati et al., 2018). Generally, MPS designs can be categorized based on the number of channels or compartments they incorporate. Single‐channel chips are frequently employed for applications involving blood flow within blood vessels and for the study of hemodynamics (Park et al., 2010). Double‐ and multi‐channel chips have been used to incorporate blood vessels and various tissues. For instance, multi‐channel MPS designs have been created to replicate the characteristics of the blood–brain barrier (BBB), intestine, lung, and tumors (Han et al., 2016; Park et al., 2019; Vatine et al., 2019). Collectively, the design of MPS exhibits significant variation, contingent upon the specific research objectives. We refer interested readers to other recent reviews that address MPS design in greater depth (Wang et al., 2020).

### 3.3 Methods of fabrication for MPS

Microfabrication is the skill of scaling down devices. An array of techniques, such as soft lithography, 3D printing, wax dipping, and laser cutting, have been extensively employed to create the desired microdevices or microstructures. Soft lithography serves as a general term encompassing a diverse array of lithography methods, including replica molding (RM), microcontact printing (μCP), micromolding in microcapillaries (MIMIC), microtransfer molding (μTM), and solvent‐assisted micromolding (SAMIM) (Pina et al., 2019). These techniques typically utilize a silicone‐based elastomer, such as PDMS, as the mold or stamp for pattern creation or transfer. Currently, the most common method for creating MPS involves the combination of soft lithography with photolithography. This approach is widely recognized for its cost‐effectiveness, reproducibility, and adaptability to a diverse array of materials compared to other methods (Cao et al., 2023). During the photolithography stage, patterned structures are created using a thin layer of photoresist (PR), which functions as a mold, enabling the production of multiple PDMS devices through the soft lithography process (Burklund et al., 2020). In the following soft lithography process, liquid PDMS is poured onto the PR mold to produce a replica, and the PDMS replica is carefully peeled from the mold and subsequently bonded to a glass slide with oxygen plasma treatment to create MPS.

In addition, 3D printing is a fast and highly accurate manufacturing technology based on a “layer‐by‐layer approach” (Tajeddin & Mustafaoglu, 2021). The emergence of 3D printing technology facilitates the fabrication of master molds commonly made by photolithography and also permits the direct creation of stamps or complex tissue and organ structures (Yazdi et al., 2016). Wax dipping is a technique used to create hydrophobic wax barriers by immersing a cellulose substrate in liquid wax, which is commonly used for fabricating paper‐based devices capable of efficiently separating blood plasma from whole blood (Songjaroen et al., 2012). A laser cutter harnesses high‐intensity CO_2_ lasers to generate hydrophilic patterns on a wide range of materials, including ceramics, plastics, papers, and glasses to create microfluidic devices featuring multilayers (Thompson et al., 2015).

In addition to the aforementioned microfabrication methods (Qamar & Shamsi, 2020), various innovative approaches have emerged, such as hot embossing (Ng et al., 2006), injection molding (Lee et al., 2018), desktop cutting (de Oliveira et al., 2017), and desktop pen‐plotter (Ghaderinezhad et al., 2017). However, due to the scope of this review, we will not delve into the specifics of these techniques.

### 3.4 MPS as a model system to study aging phenotypes across cell, tissue, and organ

The development of MPS to model aging phenotypes is still in its early stages. A 3D in vitro tissue chip model with human senescent fibroblasts and blood vessels was proposed to assess how senescent fibroblasts and aging/aged microenvironments influence the behaviors of human blood vessels (Pauty et al., 2021). A blood vessel was created in a collagen matrix containing senescent or young fibroblasts. The study discovered that senescent fibroblasts produce a higher traction force than young fibroblasts, mechanically altering the surrounding tissue, which is associated with the promotion of angiogenesis of microvessels through SASP signals. This model system provides promising potential in aging and cancer research.

**FIGURE 2:**
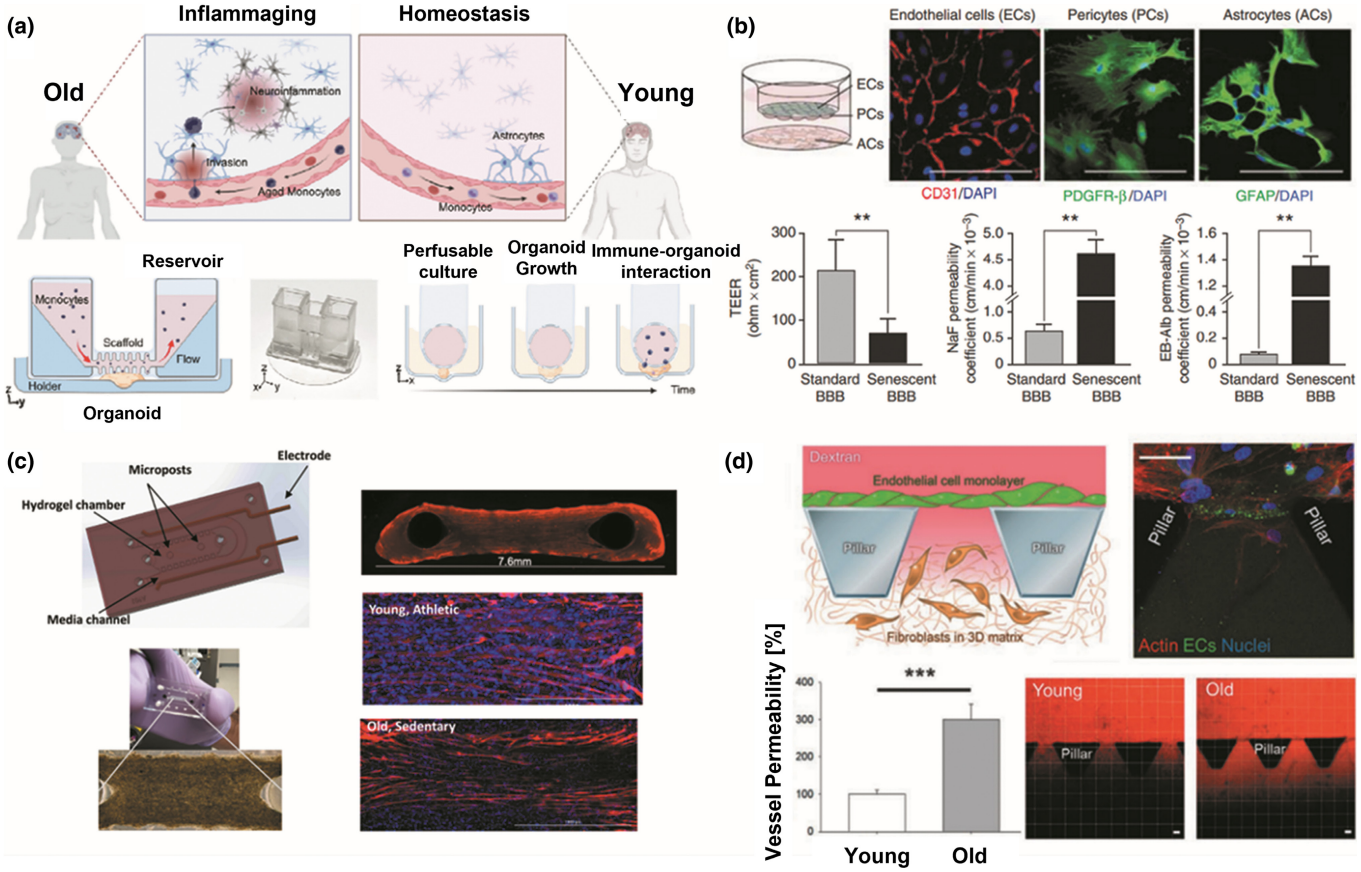
Microphysiological systems (MPS) for studying aging phenotypes. (a) MPS for immune‐driven brain aging. Primary monocytes from young and aged donors and human brain organoids were adopted and cultured in the 3D printed device to understand immune‐organoid interactions. Figure adapted with permission from (Ao et al., 2022), copyright Advanced Science. (b) In vitro triple co‐culture models of the blood–brain barrier (BBB). Endothelial cells (ECs) and pericytes (PCs) were cultured on the top and bottom sides of the semipermeable filters, while astrocytes were plated on the culture plate. Barrier functions of normal and aging BBB were quantified and compared through TEER measurement, and permeability coefficient for NaF and EB‐albumin. **p < 0.05. Figure adapted with permission from (Yamazaki et al., 2016), copyright Stroke. (c) Muscle myobundles in a microfluidic chip. Young/active and old/sedentary people‐derived muscle fibers were formed around the two posts in a microfluidic chip. The myobundles were fluorescently immunostained with myosin heavy chain antibody (MF‐20, red) and DAPI (blue). Figure adapted with permission from (Giza et al., 2022), copyright Aging Cell. (d) Microfluidic model of human endothelial cell aging. Aged endothelium was created in the microfluidic system containing pillars, and vessel permeability was measured using fluorescently‐labeled dextran. Results showed that vessel permeability in aged endothelium is substantially increased compared to that of young endothelium. ***p < 0.05. Figure adapted with permission from (Bersini et al., 2020), copyright Advanced Biosystems.

A human brain organoid MPS platform was developed using 3D printing to study the dynamics of immune‐driven brain aging (Ao et al., 2022; Figure 2a), motivated by the fact that senescent immune cells can cause a systemic aging phenotype (Yousefzadeh, Flores, et al., 2021). Primary monocytes from young (age 20–30) and old individuals (age > 60) were perfused into the platform and were analyzed for interaction with the cortical organoids. Results showed that aged monocytes tend to be more invasive with higher expression of aging markers (e.g., p16) induced in astrocytes and played an essential role in brain aging or age‐related neural diseases. This may imply that brain aging is driven by aged immune cells.

An in vitro BBB model, composed of endothelial cells, pericytes, and astrocytes from middle‐aged (senescent) and young mice, can be used for studying the development of NDs (Yamazaki et al., 2016; Figure 2b). The function of normal and aged BBB models was evaluated by quantifying the permeability coefficient with sodium fluorescein (NaF) and Evans blue (EB) albumin. The study concluded that the permeability coefficient is much higher in senescent BBB models than in standard BBB models.

A perfused MPS platform, which contains satellite cells from donors and their related skeletal muscle bundles, was developed to model muscle aging mimicking muscle disease (sarcopenia), and to investigate contractile differences between young and old adult‐derived skeletal muscle cells (Figure 2c; Giza et al., 2022). Results have shown that myobundles exhibit different aspects of contractile functions between the two groups; no synchronous contraction was found in response to electrical stimulation with lower hypertrophic potential in the old sedentary group when compared with the young active group.

To recapitulate human endothelial cell aging and investigate the integrity of the vascular barrier, a microfluidic‐based MPS model was leveraged by quantifying vessel permeability with 70 kDa dextran (Figure 2d; Bersini et al., 2020). In this study, endothelial cells from old donors (age 66) were cocultured with dermal fibroblasts from young (age 19–34) or old donors (age 63–69). The results showed that vessel permeability of aging endothelial cells was more heavily impaired when cocultured with old fibroblasts, as compared with young fibroblasts. The data implied that larger vessel leakage coupled with less vessel integrity occurred in the aging endothelial cells cocultured with old fibroblasts.

An aging cardiac tissue chip model was invented to study age‐related cardiac diseases and drug screening (Budhathoki et al., 2022). H9c2 myoblasts treated with low‐dose doxorubicin were found to drive cellular senescence with characteristics of DNA damage response, flattened and large nuclei, and elevated expression of cell cycle inhibitors, such as p53, p16^INK4a^, and ROS. Based on this aging model system, the study further developed a pathological model mimicking an infarcted aging heart via hypoxic treatment for investigating age‐related cardiovascular diseases.

While several MPS have been developed and introduced for aging research, there remains a limited number of studies aimed at comprehensively unraveling the aging physiology and phenotypes. Therefore, in the future, it is essential to conduct extensive research endeavors utilizing MPS across various aging tissue and organ systems to validate their function and effectiveness in the context of aging.

### 3.5 Application of MPS to study age‐related diseases

**FIGURE 3:**
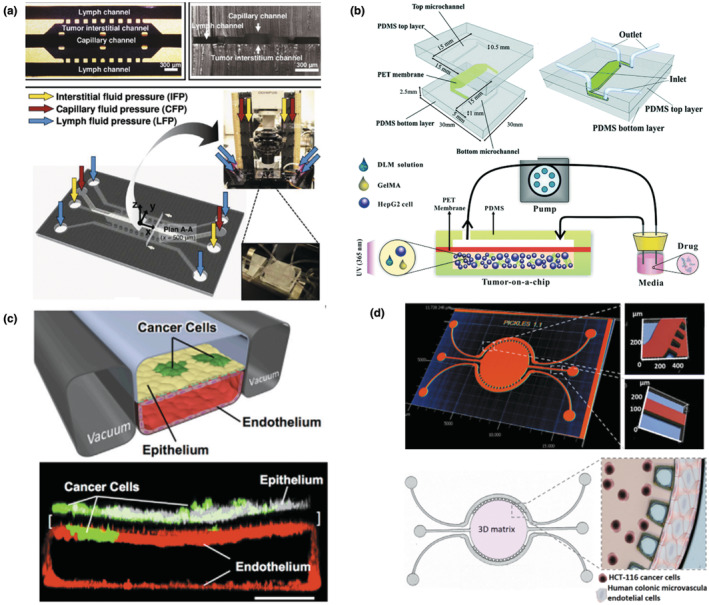
Design of diverse MPS for cancer research. (a) Mammary tumor‐on‐a‐chip. A functional unit of the mammary tumor (center channel), as well as capillary (top channel) and lymphatic vessels (two side channels), was emulated in a 3D microfluidic platform, which undergoes interstitial, capillary, and lymph fluid pressures. Figure adapted with permission from (Kwak et al., 2014), copyright Journal of Controlled Release. (b) Liver tumor‐on‐a‐chip. 3D cell culture microchip, composed of top and bottom microchannels and PET membrane, was integrated with several components derived from a decellularized liver matrix from a native liver with gelatin methacryloyl (GelMA). The hydrogel was created through photopolymerization by UV (ultraviolet) light (365 nm). Figure adapted with permission from (Lu et al., 2018), copyright Lab on a Chip. (c) Lung tumor‐on‐a‐chip. Human lung epithelial cells (white) and non‐small‐cell lung cancer cells (green) were plated on the upper surface of a porous membrane, and human microvascular endothelial cells (red) were cultured on the lower channel of the alveolus‐mimetic microfluidic chip (scale bar, 200 μm). Figure adapted with permission from (Hassell et al., 2017), copyright Cell Reports. (d) Colorectal tumor‐on‐a‐chip. Colon cancer cells (HCT‐116)‐laden Matrigel was embedded in the round central chamber (5 mm in diameter and 126 μm in depth), and human colonic microvascular endothelial cells were added in the side channels to mimic microvessels. Figure adapted with permission from (Carvalho et al., 2019), copyright Science Advances.

Various age‐related diseases have been studied via MPS, including cancer, NDs (e.g., Alzheimer's and Parkinson's disease), cardiovascular diseases or pathology (atherosclerosis and thrombosis), eye disease (e.g., macular degeneration), and musculoskeletal diseases. In this review, we mainly focus on cancer, cardiovascular diseases, and NDs, which are widely recognized as the primary causes of mortality in the United States and are among the most deadly aging‐related diseases. Cancer is a representative age‐related disease. So far, a myriad of studies have been conducted to mimic the tumor microenvironment, development, and tumorigenesis in MPS. The targeted cancers include breast (Figure 3a; Kwak et al., 2014; Lanz et al., 2017), liver (Figure 3b; Lu et al., 2018), lung (Figure 3c; Hassell et al., 2017), colorectal (Figure 3d; Carvalho et al., 2019), brain (Fan et al., 2016; Liu et al., 2010; Xiao et al., 2019), and pancreatic cancer (Mao et al., 2020; Nishiguchi et al., 2018). To understand the interaction between normal and tumor microenvironments, some studies focused on specific features (e.g., tumor vasculature) of the tumor microenvironment. For instance, Lee et al. (2014) developed a tumor angiogenesis model, where the interaction was unraveled between the tumor and stroma in a microfluidic chamber with quantification of angiogenesis.

**FIGURE 4:**
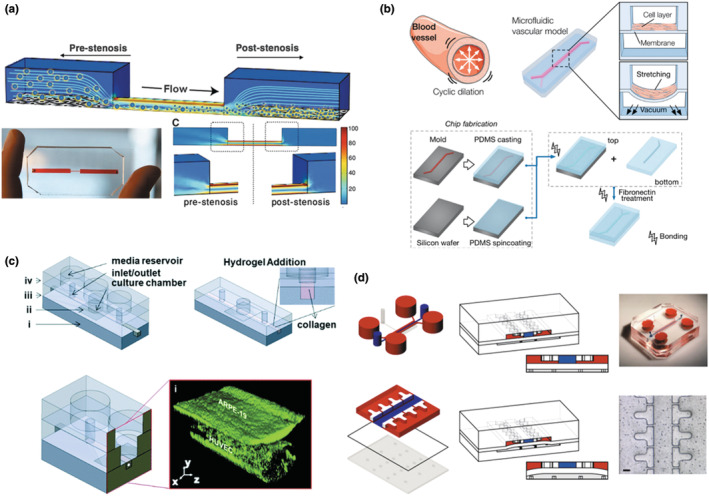
Schematics of diverse MPS for studying age‐related diseases. (a) Vascular‐on‐a‐chip to study thrombosis. A 3D microfluidic vascular stenosis model (90% lumen occlusion) with endothelial cells was fabricated to investigate shear‐induced dissociation by nanotherapeutics and nanoparticles, which are similar in size to platelets ranging from 1 to 5 μm in diameter. The shear rate in the region of the stenosis was increased to ~100,000 s^−1^ from 1000 s^−1^ of a physiological shear rate. Figure adapted with permission from (Korin et al., 2012), copyright Science. (b) Progeria‐on‐a‐chip. The microfluidic vascular model is comprised of two compartmentalized channels where senescent aortic smooth muscle cells are cultured on top of the deformable PDMS membrane in the upper channel and undergo cyclic strain mimicking pulsatile blood flow. Figure adapted with permission from (Ribas et al., 2017), copyright Small. (c) MPS for the outer blood‐retinal barrier to study macular degeneration. The microscale system contains (i) a bottom compartment, (ii) a polyester membrane with pores, (iii) an open‐top culture chamber, and (iv) a top compartment. The microvessel surrounded by collagen hydrogel I was created using blunt needles, and human endothelial cells and retinal pigment epithelial cells were cocultured. Figure adapted with permission from (Arik et al., 2021), copyright Lab on a Chip. (d) Cartilage‐on‐a‐chip model to study osteoarthritis. Two PDMS chambers were fabricated and separated by a PDMS membrane. The chondrocyte‐laden PEG polymer solution was injected into the central upper chamber (blue). By pressurizing the bottom chamber, mechanical compression can be generated by the deformation of the PDMS membrane (Scale bar, 100 μm). Figure adapted with permission from (Occhetta et al., 2019), copyright Nature Biomedical Engineering.

Uncovering the molecular and cellular mechanisms of cardiovascular diseases is essential to establish an effective treatment. MPS have been applied to develop in vitro vascular models (aka, vascular‐on‐a‐chip) and heart models (aka, heart‐on‐a‐chip) to examine the pathogenesis of cardiovascular diseases. These pathogeneses and diseases include arteriovenous thrombosis/stenosis (Figure 4a; Conant et al., 2011; Korin et al., 2012; Zheng et al., 2012), atherosclerosis (DeVerse et al., 2012), myocardial infarction (Ren et al., 2013), cardiac hypertrophy, or heart failure (Dirkx et al., 2013; Tan et al., 2002).

NDs have been studied with different forms of MPS. To investigate the pathological mechanisms of NDs with the interfaces of neural tissue, “Neural MPS” was established with various cell sources, biomaterials, and fabrication techniques (Osaki et al., 2018; Pamies et al., 2017). Of note, neural MPS have great potential to study the mechanisms of Alzheimer's or Parkinson's disease. For example, a 3D neural cell culture system was developed by two groups, where the pathology of Alzheimer's disease was recapitulated with a human neural progenitor cell line (Choi et al., 2014; Kim et al., 2015). They showed through the 3D culture system that amyloid‐β is highly deposited in ECM by mutations of Familial Alzheimer's disease (FAD) in presenilin 1 and β‐amyloid precursor protein. To mimic and study Parkinson's disease, Kane et al. (2020) developed a 3D microfluidic system from patient‐derived neuronal cells, where neuroepithelial stem cells were differentiated into dopaminergic neurons.

Hutchinson–Gilford progeria syndrome (HGPS) is a premature aging disorder triggered by a point mutation in the lamin A gene. This mutation leads to accelerated cardiovascular disease, among other age‐associated abnormalities (Goldman et al., 2004; Ribas et al., 2017). Ribas et al. (2017) developed a progeria‐on‐a‐chip system to understand the effects of physiological and pathological stretching conditions on vascular aging (Figure 4b). Intriguingly, smooth muscle cells derived from human induced‐pluripotent stem cells (hiPSCs) of HGPS patients displayed an increased level of inflammation and DNA damage in response to strain compared to healthy donors.

Several MPS have been constructed to recapitulate ocular diseases, such as glaucoma, high myopia, and diabetic eye diseases. To understand age‐related macular degeneration, Arik et al. (2021) invented a microfluidic OoC system to mimic the outer blood‐retinal barrier using human‐induced pluripotent stem cell‐derived retinal pigment epithelium (hiPSC‐RPE) and human‐induced pluripotent stem cell‐derived endothelial cells (hiPSC‐ECs) (Figure 4c). The level of cell–cell adhesion was quantified by immunocytochemistry, and the function of the endothelial barrier was quantified with the permeability of fluorescently‐labeled dextran. In addition to the aforementioned diseases, numerous types of MPS have been developed to emulate diverse age‐related musculoskeletal diseases, such as neuromuscular disease (Park et al., 2013; Zahavi et al., 2015), sarcopenia (Sharples et al., 2012), and osteoarthritis (Figure 4d; Occhetta et al., 2019).

As shown above, a number of MPS have been developed to study aging‐related diseases. However, the majority of these studies have predominantly concentrated on individual tissues or organs. To propel our understanding further, future research should strive to integrate multiple organs or tissues into a single chip, thereby constructing a more complex multi‐organ chip model, ultimately realizing the concept of a “Human Aging‐on‐a‐Chip.”

### 3.6 Application of MPS to drug screening

MPS are commonly employed for drug screening, assessing drug efficacy and toxicity. Currently, a wide array of drugs, ranging from antipsychotics and analgesics to anticancer and senolytic drugs, have been utilized through MPS for screening tests (Wang et al., 2023). However, despite the extensive use of MPS for a variety of drug tests, drug screening assays in MPS for human aging research have been notably limited. The most frequently examined organs for drug screening encompass the heart, liver, kidney, and brain.

The heart functions as a dynamic mechanical system, playing a crucial role in pumping blood throughout the entire circulatory system. In heart MPS, various drugs, such as verapamil (Zhang, Wang, et al., 2016), isoproterenol (Agarwal et al., 2013), doxorubicin (Zhang, Arneri, et al., 2016), terfenadine (Kujala et al., 2016), and fexofenadine (Kujala et al., 2016), have been used for both drug efficacy and cardiotoxicity screening. An illustrative instance involves verapamil, an antiarrhythmic drug, which was introduced and evaluated in a heart MPS constructed using primary neonatal rat cardiomyocytes (Zhang, Wang, et al., 2016). The findings demonstrated a decrease in the beating rate and contractility of cardiomyocytes. In another study, doxorubicin, a chemotherapeutic drug, was assessed in a heart MPS comprising human iPSC‐derived cardiomyocytes and endothelial cells, with the beating rate of cardiomyocytes quantified via optical microscopy (Zhang, Arneri, et al., 2016).

The liver occupies a central position in drug metabolism and plays a pivotal role in detoxification processes (Lee, 2017). To date, several liver MPS models have been developed to explore hepatotoxicity through the use of various drugs such as acetaminophen (APAP, an analgesic and antipyretic drug), chlorpromazine (an antipsychotic drug), and tacrine. As an illustration, a microfluidic chip utilizing hepatocellular carcinoma (HepG2/C3A) cells was developed to elucidate the APAP injury pathway (Jaeschke et al., 2021). Various functional markers, including lipid peroxidation, metabolic activity, cell density, and viability in the presence of APAP, were systematically investigated and characterized (Knowlton & Tasoglu, 2016; Prot et al., 2012).

The kidney serves as a pivotal regulator of blood filtration and urine production (Rehberg, 1926). Furthermore, its role is closely intertwined with metabolic processes and drug clearance (Wang et al., 2023). Various models of kidney or tubule MPS have been put forth to explore and forecast nephrotoxicity, encompassing the use of a range of drugs, such as cyclosporine A, gentamicin, cisplatin, tenofovir, polymyxin B, and tobramycin (Kim et al., 2016; Vormann et al., 2021; Yin et al., 2020). Multiple kidney and nephrotoxicity markers, such as epithelial barrier function, cell polarity, membrane integrity, and mitochondrial function, were evaluated in the MPS after drug induction (Faria et al., 2019). For instance, a 3D bioprinted kidney proximal tubule chip was developed to replicate the structure and function of proximal tubules (Homan et al., 2016), with cyclosporine introduced into the MPS. The findings demonstrated a significant impairment in epithelial barrier functions following the drug addition.

The brain, which is the most complex organ in the human body, governs functions such as thought, memory, emotion, touch, motor skills, vision, and more (Haynes & Rees, 2006). It comprises three primary regions—the forebrain, midbrain, and hindbrain—and a diverse array of cells, including nerve cells, microglial cells, astrocytes, and more. Consequently, the development of brain MPS and drug screening tests is still in its early stages, primarily due to the formidable challenge of replicating the brain's intricate structure and functionality (Harberts et al., 2020). Presently, the majority of studies in the field of MPS related to the brain have been concentrated on replicating specific tissue components, notably the BBB and the neurovascular unit, in order to assess neurotoxicity and drug transport (Wang et al., 2023). For example, a neurovascular unit MPS, in conjunction with two BBB chips, was engineered to examine the metabolic function of brain vessels by introducing methamphetamine (a psychoactive drug) into the vessels (Maoz et al., 2018). An in vitro BBB model‐on‐chip, composed of PDMS, electrode layers, and polycarbonate membranes, was introduced for the prediction of drug transport using different‐sized dextran and propidium iodide (Booth & Kim, 2012). These MPS devices hold significant promise for applications in aging research, related to drug delivery, efficacy, toxicity assessment, and drug transport.

Senolytics, such as dasatinib, quercetin, fisetin, and navitoclax, are pharmaceutical agents to selectively remove senescent cells, recognized as a pivotal marker of the aging process (Kirkland & Tchkonia, 2020). However, the utilization of senolytic drugs in drug screening assays within MPS devices remains extremely limited. Recently, Mourad et al. conducted a drug efficacy assessment involving the combination of senolytic agents, dasatinib and quercetin, within a cardiac fibrosis‐on‐a‐chip model. This model integrated human‐induced pluripotent stem cell‐derived cardiomyocytes and cardiac fibroblasts (Mourad et al., 2023). The research findings demonstrated that the application of senolytic drugs led to enhancements in functional properties, including increased contractility, reduced passive tension, and a decrease in senescence‐related gene expression.

Collectively, MPS devices have been widely leveraged in various drug screening applications across different organs and tissues. Nonetheless, the integration of MPS with drug screening in human aging models remains significantly unexplored. In addition to evaluating interventions that mitigate age‐related biological changes, such as senolytics, MPS could also address the vital need for model systems that can recapitulate how age‐related cellular and physiological changes affect the toxicity and efficacy of existing drugs, and to model common clinical states of aging such as polypharmacy and multimorbidity that can further affect drug safety (Huizer‐Pajkos et al., 2016; Lavan & Gallagher, 2016). Despite their limited current use in age‐related contexts, the studies mentioned earlier, conducted for drug screening purposes, exemplify the promising potential and advantages of utilizing MPS in the realm of human aging research.

## 4 LIMITATIONS AND FUTURE PERSPECTIVES FOR AGING RESEARCH

**FIGURE 5:**
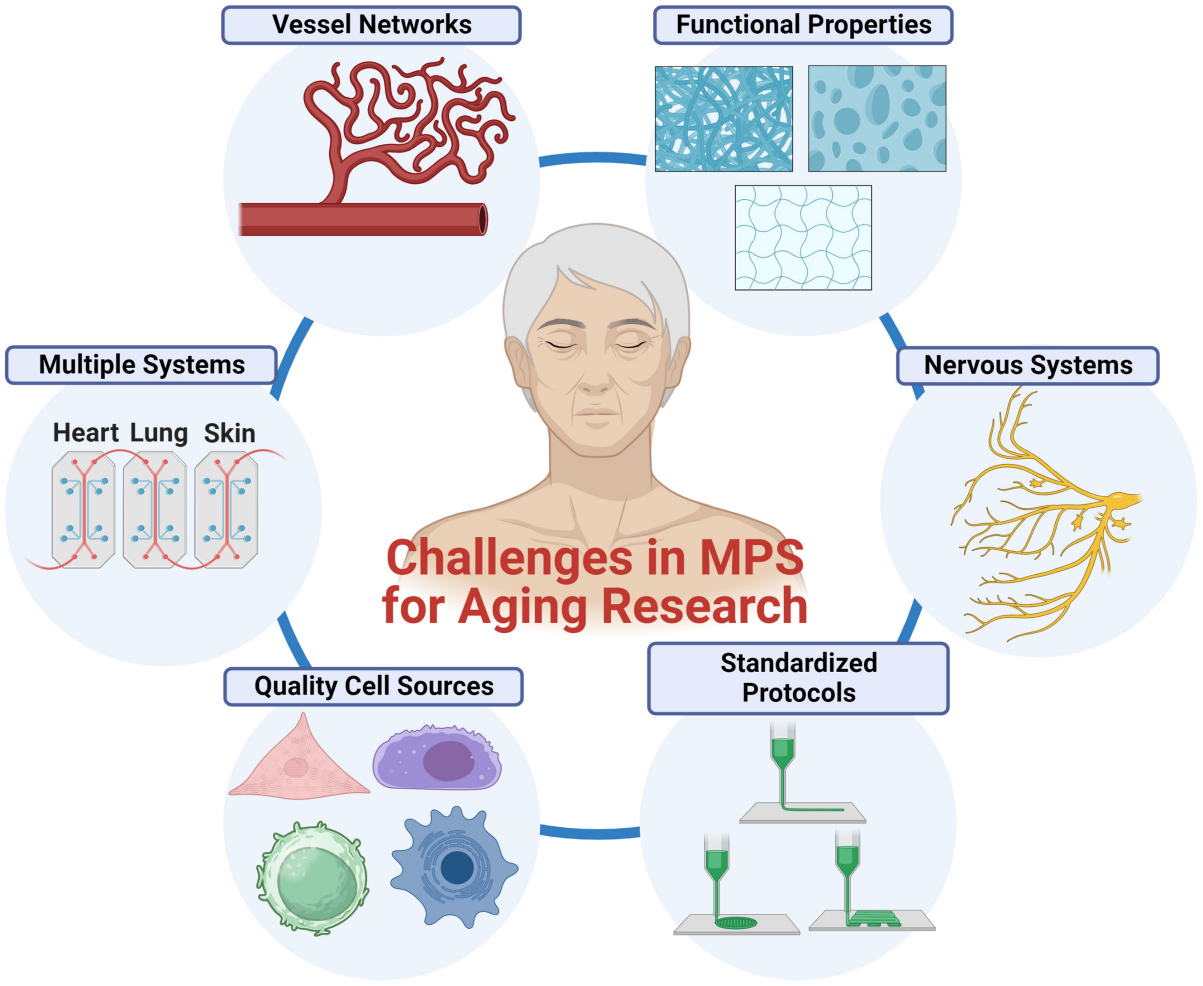
Challenges in MPS for aging research. Key challenges encompass the development of vascularization, integration of multi‐MPS platforms for various organs and tissues (e.g., human‐on‐a‐chip systems), procurement of high‐quality human cells representing healthy aging/aged states, establishment of standardizing protocols, replication of nervous system components, and recapitulation of functional properties including mechanical, structural, and transport properties.

Significant progress has been made in MPS for aging research, but many barriers and limitations remain to be addressed and considered (Figure 5). First, modeling functional vascular networks to provide oxygen and nutrients to the tissues is one of the limiting factors in MPS for the study of age‐related phenotypes, or any other physiological system (Auger et al., 2013). In addition, since aging‐associated vascularization may be highly implicated in changes in the exchange of hormones, metabolites, and cytokines, it is essential to consider mimicking appropriate aging and young vasculatures and incorporate them into tissues and organs in MPS (Villeda et al., 2014). How to accurately measure the vessel's functional properties, such as vascular permeability or hydraulic conductivity, is another integral part to be considered after the formation of vessel networks. These properties are the key to demonstrating the degree of diffusion and transport of nutrients and oxygen across the vessel. Notably, these properties are also well‐known to change with age.

Second, MPS studies did not pay enough attention to physical properties, including mechanical, structural, or transport properties of cells and tissues during aging. In fact, those properties should match the actual properties in physiological aging conditions. Prior research has shown that different types of tissues or cells exhibit different tendencies to change in mechanical properties with age. For example, some tissues, such as corneal or skin tissue, display an increase in stiffness during aging, whereas other tissues, including bone tissue, show a decrease. As such, matching the functional physical properties can significantly advance the understanding of the physiological relevance of MPS. Structural properties such as ECM fiber orientation in tissue are also substantially altered in the aging process. Studies have revealed that the ECM fibers of dermal skin tissue tend to be more aligned in aging/aged tissue while maintaining more isotropic networks in young tissue, thus inducing abnormal mechanical and transport properties (Kaur et al., 2019; Park, 2022). Hence, these factors should be considered in future studies when MPS are designed for aging research.

Third, most of the research using MPS has been focused on a single tissue or organ. However, in natural physiological systems, all organs and tissues are highly interconnected in the body and interact with each other. Furthermore, the aging process can be driven or even accelerated by the combined effects of these systems, rather than that of an individual system. MPS emulating multiple organs were developed to study the interaction of organs, mainly focusing on drug interactions (Vernetti et al., 2017), which provides insight and potential for drug discovery in aging research. However, to our knowledge, aging research with MPS is extremely rare with multi‐tissue and multi‐organ systems, except for the incorporation of vasculature into tissues or organs. One of the primary reasons is that it is challenging to develop a single, universal medium that can be used across multiple tissues and organs. Incorporating multiple tissues or organs is needed to mimic the complex physiological aging conditions and thus help understand exact age‐related physiology and pathology. Such multiple tissue or organ systems may hold particular promise for the mechanistic study of the “integrative” hallmarks of aging such as chronic inflammation, dysbiosis, stem cell exhaustion, and altered intercellular communication, and help bridge the gap between intracellular aging processes and systemic physiologic dysfunction affecting older adults (Acun et al., 2017; López‐Otín et al., 2023).

Fourth, many biological functions in various tissues and organs, such as cardiac muscle, glands, or liver, are controlled by the nervous system, and thus, innervation is another key factor to be considered in the aging model system (Park, Lee, et al., 2020). Many studies have been conducted to investigate functions of innervation in different tissues using synaptic, neuromuscular junction (NMJ), or neuroeffector junction (NEJ) innervation‐on‐a‐chip. These have employed electrode tools, including nanoelectrode or microelectrode arrays (Dai et al., 2016; Guo et al., 2017; Natarajan, 2013; Tian et al., 2012).

Fifth, another challenge is to obtain high‐quality human cells or aged human cells at different stages. As such, studies have used established cell lines that are highly proliferative, easier to manage, and easier to culture compared to primary cells. However, the cell lines may not have the essential markers and functions observed in vivo. To increase the accuracy of data, it is essential to utilize primary cells directly derived from younger and older people for aging research. Further work should also explore methods to recapitulate in iPSC‐derived cells the phenotypes of primary cells derived from older adults—potentially utilizing known aging mechanisms such as DNA damage, epigenetic alterations, or induced mitochondrial dysfunction. Considerable caution is required in this endeavor, as discussed below.

Sixth, standardized protocols for MPS have currently been poorly established for mimicking many tissues and organs, which results in high variation and inconsistency in the experimental data from one group to another. Thus, well‐established methods are required to set the standard for reproducibility in the field.

Lastly but most importantly, most of the MPS may not directly reflect the aging process in humans. Most of the aging research has been focused on mimicking age‐related diseases to find an effective treatment for those diseases, rather than aging phenotypes in biological systems. But aging phenotypes are significant to evaluate the aging process, which will eventually provide the cellular and molecular mechanisms for aging pathologies and diseases. Understanding such physiological changes during aging can provide a seminal clue to preventing the occurrence and development of deadly age‐related diseases. Hence, further research with MPS is needed to recapitulate complex and dynamic aging microenvironments composed of aging cells, tissues, or age‐related molecules. Finally, despite much progress in MPS, there is still a limitation in translating the results from MPS into clinical settings due to a huge physiological gap between humans and MPS. Therefore, we should not overgeneralize or misinterpret the results unless the data are validated by different techniques from different groups.

It should be noted that different molecular and physiologic aging pathways may drive distinct cell, tissue, and organ phenotypes. These distinct aging phenotypes may be more or less relevant for a particular MPS system intended to model normal aging, physiologic resilience, or an age‐related disease. To take one example of this complexity, multiple forms of cellular senescence have been demonstrated depending on cell type, physiologic context, and the factors used to induce senescence: repeated cell replication, UV (ultraviolet) light, oxidative stress, disturbed flow, drugs, microgravity and so on (Acun et al., 2017; Kirschner et al., 2020). However, the current review was primarily focused on natural or chronological aging. Hence, it is crucial to be aware that the features of aging demonstrated by MPS that utilize primary cells from older adults may differ from systems in which aging mechanisms are experimentally induced. In the case of cellular senescence, studies have shown that stress‐induced senescence due to disturbed flow or cancer drugs results in stronger expression of SASP, excessive ROS, higher levels of telomere DNA damage, shelterin complex dysfunctions, and senescence‐associated reprogramming/stemness, as compared to natural aging (Dominic et al., 2020). The relevance of MPS that incorporate aging phenotypes extends beyond models of healthy aging states or age‐related diseases. Such MPS could be used to understand how aging phenotypes interact with dynamic stressor responses and physiological resilience. Nowadays, as the possibility of space travel increases, many studies have been conducted on how space environments, such as a vacuum and zero‐ or microgravity, influence human physiology and diseases (Low & Giulianotti, 2020). It is hypothesized that spaceflight and microgravity may accelerate the development of aging phenotypes (e.g., elevated bone resorption, muscle wasting, and skeletal muscle atrophy). People in space may develop age‐related diseases (e.g., osteoporosis) at a younger age, due to dramatic changes in human physiology and biology (Lee et al., 2022; Sharma et al., 2022). For example, muscular atrophy, defined as a decrease in muscle mass, is the most noticeable phenomenon in spaceflight, which seems to be comparable to muscular atrophy in natural aging (Lee et al., 2022). Another study showed that increased oxidative stress and poor stress response could cause skeletal muscle atrophy during mechanical unloading, which often happens with spaceflight (Lawler et al., 2021). Therefore, which aging mechanisms are selected to induce the aging phenotype under investigation will be another significant factor when designing and developing aging model systems using MPS in future studies.

Acknowledging these limitations and cautions, MPS provide valuable tools for research in aging, with the ability to control chemical gradients, explore cell migration, identify cell‐secreted molecules, and conduct electrical or electrochemical measurements. Cell migration plays a pivotal role in various biological processes, encompassing embryonic development, wound healing, immune responses, and cancer metastasis (Sala et al., 2022). Many instances of cell migration are orchestrated by chemoattractants, cytokines, and other signaling molecules. MPS platforms typically composed of PDMS (Mak et al., 2011; Spuul et al., 2016; Zhou et al., 2021), hydrogels (Anguiano et al., 2020; Ayuso et al., 2017; Huang et al., 2015), glasses (Sima et al., 2020), and/or photopolymers (Olsen et al., 2013; Tayalia et al., 2008) have been proposed to investigate cell migration and chemical gradients. As an example, Rolli et al. (2010) developed a straightforward high‐throughput microfluidic system based on PDMS. This system featured two reservoirs interconnected by multiple channels. The research aimed to quantify the motility velocity of human pancreatic epithelial cancer cells within both microchannels and on a culture dish, simulating confined and unconfined conditions. The findings revealed that cells within the microchannels, representing a confined environment, exhibited faster and more consistent sliding movement in comparison to their unconfined counterparts.

Irimia et al. (2007) conducted an experimental study to explore the impact of chemical gradients on cell migration under highly confining conditions. Their research involved the use of various drugs, including both attractants and inhibitors, to investigate leukocyte migration. The experimental device featured a central region flanked by two side channels, allowing for direct contact between drug and buffer solution fluxes within the laminar flow regime. This setup generated a linear chemical gradient across the area where cell migration was studied. Tong et al. (2012) engineered a microfluidic channel that facilitated the creation of a stable concentration profile, enabling the study of cellular behavior across various cell lines. These cell lines included human osteosarcoma cells (HOS), human breast adenocarcinoma cells (MCF‐7, MDA‐MB‐231), and non‐tumorigenic mammary epithelial cells (MCF‐10A). Building on these prior investigations, potential applications in aging research could involve examining the migration and motility of both tumor cells and skin cells within MPS featuring aging tissue mechanical or structural characteristics, such as heightened stiffness or aligned collagen or ECM fiber networks by inducing chemical gradients. Thus, researchers can gain insights into the impact of aging on processes like cancer metastasis and wound healing.

Numerous investigations have employed MPS to identify the presence of cell‐secreted molecules. For instance, Son et al. (2017) pioneered the development of an MPS that incorporates a microfluidic device designed to identify the secretion of transforming growth factor (TGF)‐β1 and hepatocyte growth factor (HGF) by primary hepatocytes using a fluorescent microbead‐based assay. Droplet‐based MPS platforms have been proposed for the purpose of detecting molecular or cytokine secretions from individual cells. Konry et al. (2011) detected the secretion of interleukin‐10 (IL‐10) by T cells, through a method involving the encapsulation of primary T cells within aqueous droplets. These droplets contained beads conjugated to anti‐IL‐10 antibodies. Such innovative systems can offer potential, versatile applications, enabling the separation and isolation of aging cells based on their specific cytokine expression profiles or levels (Frenzel & Merten, 2017; Xi et al., 2017). Furthermore, with regard to the potential future of aging research, it is conceivable to incorporate aging or senescent cells within MPS to investigate whether cell‐secreted molecules, such as SASP factors, have the capability to impact the behavior of neighboring cells or the molecules secreted by those neighboring cells.

MPS platforms have also been integrated with electrochemical‐sensing devices for the purpose of quantifying the concentrations of a wide range of cell‐secreted substances. These substances encompass glucose (Misun et al., 2016), oxygen (Moya et al., 2018), reactive oxygen species (ROS) (Cheah et al., 2010), or other related biomarkers (Shin et al., 2016, 2017) typically generated in damaged tissues. As one of the examples, Shin et al. introduced a microfluidic‐electrochemical sensor unit that combines electrochemical sensors with a microfluidic bioreactor. This system is designed for the detection of creatine kinase (CK)‐MB, which is associated with the dysfunction of cardiac organoids, as described in their studies (Shin et al., 2016, 2017). As such, electrochemical techniques can offer the means to quantify and characterize the activities of various aging‐related genes (such as CK‐MB, p21, and p53) or molecules (such as telomere erosion and DNA damage response) that provide insights into the functionality of certain tissues or organs such as cardiac or neuromuscular tissues and organs. These assessments using electrochemical methodologies are invaluable for evaluating the aging process and aging‐related pathologies.

Within the realm of scientific literature, numerous reviews have delved into the exploration of chemical gradients, cell migration, cell‐secreted molecules, and electrical or electrochemical measurements within MPS platforms. Specifically, we direct the reader to the comprehensive work of Sala et al. (2022) and Hiramoto et al. (2019) for a more profound and insightful exploration of this subject matter.

In this comprehensive review, we delved into the use of MPS in human aging research. First, we introduced the current status of MPS, elucidating their unique features by comparing them with other model systems. Next, we examined the vast spectrum of aging‐related biological changes in different scales and highlighted how aging biology connects to aspects of current or future MPS design. We subsequently addressed the previous and current application of MPS in understanding aging‐related phenotypes and diseases, as well as their potential role in drug screening for aging research. Finally, we explored the present limitations and future directions of MPS in the field of aging research. Despite the previously stated limitations, MPS can be powerful model systems for understanding the impact of aging on human physiology and pathology. It overcomes the ethical issues inherent in animal models. MPS provide a reproducible and high‐throughput system for modeling aging and human diseases and has the potential to simulate human tissues with fidelity. Ultimately, this system will get us closer to swiftly finding therapeutic solutions for various age‐related pathologies and diseases.

## AUTHOR CONTRIBUTIONS

S.P., J.C., and P.G. conducted an extensive literature review and collaboratively prepared the initial draft of the manuscript. S.P., T.C.L., B.D., and D.‐H.K. wrote the original draft of the article with input from all co‐authors. S.P. prepared the figures. All co‐authors reviewed and edited the article.

## FUNDING INFORMATION

This study was supported by the National Institutes of Health (NIH) grants K25AG070286 (S.P.), UH3TR003519, R01HL156947, R01 HL164936, R01HL146436, R01HL164936, and UH3TR003271 (D.‐H.K.).

## CONFLICT OF INTEREST STATEMENT

D.‐H.K is a co‐founder and scientific advisory board member at Curi Bio, Inc.

## Data Availability

Data sharing is not applicable to this article as no new data were created or analyzed in this study.
